# Aurora-a confers radioresistance in human hepatocellular carcinoma by activating NF-κB signaling pathway

**DOI:** 10.1186/s12885-019-6312-y

**Published:** 2019-11-08

**Authors:** Ze-Tian Shen, Ying Chen, Gui-Chun Huang, Xi-Xu Zhu, Rui Wang, Long-Bang Chen

**Affiliations:** 1 Department of Radiation Oncology, Jinling Hospital, Nanjing Medical School University, Nanjing, Jiangsu China; 2 Department of Medical Oncology, Jinling Hospital, School of Medicine, Nanjing University, Nanjing, Jiangsu China; 3 Department of Medical Oncology, Jinling Hospital, Nanjing Medical School University, Nanjing, Jiangsu China

**Keywords:** Aurora-a, Hepatocellular carcinoma, Radioresistance, NF-kappaB, Apoptosis

## Abstract

**Background:**

Radiotherapy failure is a significant clinical challenge due to the development of resistance in the course of treatment. Therefore, it is necessary to further study the radiation resistance mechanism of HCC. In our early study, we have showed that the expression of Aurora-A mRNA was upregulated in HCC tissue samples or cells, and Aurora-A promoted the malignant phenotype of HCC cells. However, the effect of Aurora-A on the development of HCC radioresistance is not well known.

**Methods:**

In this study, colony formation assay, MTT assays, flow cytometry assays, RT-PCR assays, Western blot, and tumor xenografts experiments were used to identify Aurora-A promotes the radioresistance of HCC cells by decreasing IR-induced apoptosis in vitro and in vivo. Dual-luciferase reporter assay, MTT assays, flow cytometry assays, and Western blot assay were performed to show the interactions of Aurora-A and NF-κB.

**Results:**

We established radioresistance HCC cell lines (HepG2-R) and found that Aurora-A was significantly upregulated in those radioresistant HCC cells in comparison with their parental HCC cells. Knockdown of Aurora-A increased radiosensitivity of radioresistant HCC cells both in vivo and in vitro by enhancing irradiation-induced apoptosis, while upregulation of Aurora-A decreased radiosensitivity by reducing irradiation-induced apoptosis of parental cells. In addition, we have showed that Aurora-A could promote the expression of nuclear IkappaB-alpha (IκBα) protein while enhancing the activity of NF-kappaB (κB), thereby promoted expression of NF-κB pathway downstream effectors, including proteins (Mcl-1, Bcl-2, PARP, and caspase-3), all of which are associated with apoptosis.

**Conclusions:**

Aurora-A reduces radiotherapy-induced apoptosis by activating NF-κB signaling, thereby contributing to HCC radioresistance. Our results provided the first evidence that Aurora-A was essential for radioresistance in HCC and targeting this molecular would be a potential strategy for radiosensitization in HCC.

## Background

Hepatocellular carcinoma (HCC) is one of the most common malignancies encountered in clinical practice, ranking second in mortality rate around the world [1]. Although surgical resection has long been the first choice for the treatment of HCC, less than 30% of HCC patients are indicated for surgery. Non-surgical treatments (local ablation therapy, molecular-targeted therapy, radiotherapy, etc.) for HCC, have practically improved the quality of life of HCC patients, but the overall therapeutic outcomes are still not significantly improved [2]. Recently, with the development of three-dimensional conformal radiation therapy, stereotactic radiotherapy, proton radiation therapy, heavy ion radiation therapy, and other technologies, the volume of irradiated normal liver tissue has been greatly reduced, and radiotherapy has gradually become an effective means for the treatment of HCC [3, 4]. However, radiotherapy failure remains a significant clinical challenge due to the development of resistance in the course of treatment. The development of radiotherapy resistance is complicacy associated with a variety of biological factors, while the specific mechanism remains unclear. Therefore, elucidating the molecular mechanism involved in radioresistance will help to develop new therapeutic targets to overcome the radioresistance to achieve better therapeutic outcomes in HCC patients.

Aurora kinase family, including Aurora-A, B and C, belongs to the serine/threonine kinases and involved in cell mitosis [5]. Among these three molecules, Aurora-A is also called STK15, which is the most important kinase molecule in the Aurora family, composed of an N-terminal β-chain domain and a C-terminal α-helix domain. Aurora-A plays an important role in the process of normal cell mitosis and the development and progression of tumors [6, 7]. Several studies have indicated that Aurora-A was highly expressed in a variety of malignant tumors, including HCC [8]. Also, our previous study confirmed that clinical stage and lymphatic metastasis of HCC patients could effectively affect the expression level of Aurora-A, and its inhibition could significantly reverse malignant phenotypes of HCC cells [9, 10]. Importantly, we testified that overexpression of Aurora-A could induce chemoresistance in HCC by activation of nuclear factor-kappa B (NF-κB)/microRNA-21/PTEN pathway [11]. Nevertheless, the mechanism of Aurora-A in HCC radioresistance is still unclear and needs to be elucidated. NF-κB pathway activations are considered to play roles in the development of radioresistance in many types of tumor cells [12]. Recent studies have shown that NF-κB could up-regulate the expression of downstream target genes such as c-Myc and cyclin D1 to directly promote cell proliferation and inhibit cell apoptosis [13]. Also, NF-κB could inhibit the mitochondrial-dependent apoptosis pathway which is regulated by the BCL-2 family composed of both anti-apoptotic and pro-apoptotic proteins residing within and outside the mitochondrial membrane [14]. Under the action of ionizing radiation, NF-κB is overactivated, thereby upregulating certain inhibitor of apoptosis protein such as BCL-XL [15]. These anti-apoptotic proteins not only inhibit cell release of pigment C but also inhibit caspase 9 activation, eventually leading to radiotherapy resistance. Since Aurora-A could cause abnormal activation of NF-κB and further activate the NF-κB signaling pathway [16], we hypothesized that Aurora-A might play an important role in inhibiting cell apoptosis via regulating NF-κB to contribute to radioresistance in HCC cells.

To test this possibility, we established radio-resistant HCC cell lines to explore the roles of Aurora-A in the acquired radioresistance of HCC cells. We further examined the relationship between Aurora-A and activation of the NF-κB pathway and searched for downstream genes activated upon this interaction.

## Methods

### Cell culture and establishment of radio-resistant HCC cell lines

A parental human HCC cell line (HepG2) was purchased from Shanghai Institute of Cell Biology, Chinese Academy of Sciences. The cells were cultured in Dulbecco’s modified Eagle medium (GIBCO-BRL) containing 10% fetal bovine serum, 100 U/mL penicillin, and 100 μg/mL streptomycin at 37 °C with 5% CO_2_. A linear accelerator was used to irradiate HepG2 cells in the logarithmic phase. The initial dose was 2.0 Gy. After two repetitions, the dose was gradually increased to 4.0 Gy, 6.0 Gy, 8.0 Gy, 10.0 Gy, etc. The cells were irradiated twice with each dose until reaching the final dose of 60 Gy. The last surviving cells were used to establish radioresistant cell lines, designated HepG2-R.

### RT-PCR

The total RNA of HCC cells in each group was extracted separately. Using SYBR® Green I dye as the detection signal and GAPDH as an internal reference, the expression levels of Aurora-A were measured.

### Western blotting assay

Western Blotting was carried out as previously reported [11]. In brief, collected cells were lysed, and the total proteins were separated in 10% SDS-acrylamide gel. Then, the separated proteins were transferred to a polyvinylidene fluoride membrane (American Thermal Science). Aurora-A, IκBα, p65, Bcl-2, Mcl-1, cleaved PARP, and cleaved caspase-3 antibodies (Univ-bio Inc., Shanghai, China) were applied for the detection of protein expression. The amount of protein expression was determined using mouse anti-GAPDH monoclonal antibody (Univ-bio Inc., Shanghai, China). Lab Works™ Image Acquisition was used to quantify band intensities, so as Analysis Software (UVP, Upland, CA, USA).

### Establishment of stable cell lines

Lenti-shAurora-A (Lv-shAuro), Lenti-Aurora-A (Lv-Auro) and control lentiviral vectors were purchased from GenePharma Co., Ltd. (Shanghai, China). After transfection, cells were exposed to 4.0 μg/mL puromycin. Via selection stress, stably transfected HCC cell lines including Aurora-A-overexpressing and Aurora-A-knockdown cell lines were obtained and named HepG2-R/shAuro (or HepG2-R/shcontrol) and HepG2/Auro (or HepG2/control), respectively. Monoclonal cell lines were generated from single clones.

### Colony formation assay

The cells were seeded at a density of 800–2000 cells/well in a six-well plate containing culture medium. After 24 h incubation, the cells were irradiated with X-rays and then placed in an incubator for 14 days. The cells were then fixed in 4% paraformaldehyde and stained with crystal violet. The colonies were dried and visually counted.

### In vitro radiosensitivity assay

First, we prepare single-cell suspensions and disperse them in a 96-well plate and addition of 3-(4,5-Dimethyl-2-thiazolyl)-2,5-diphenyl-2H-tetrazolium bromide (MTT) assay (Sigma, USA) solution (0.5 mg/ml) then treated with various doses of IR, then hatch for 4 h, add 100 ul of extraction buffer to each medium. After one night of incubation, the absorbance was measured through a microplate reader (Bio-Rad, Model 680) at 490 nm.

### In vivo radiosensitivity assay

Animal experiments are conducted according to institutional guidelines (the Section of Comparative Medicine, Jinling Hospital, Nanjing, China). Female BALB/c nude mice between 5 and 6 weeks old were obtained from the Animal Core Facility of Nanjing Medical University (Nanjing, China) and housed in laminar flow cabinets under specific pathogen-free conditions. Stably transfected hepatocytes were suspended in 100 μl PBS and subcutaneously injected into the right flank of the female BALB/c nude mouse. At seventh-day post tumor cell injection, the tumors were treated with 8.0 Gy IR. Tumor growth was examined weekly for at least 6 weeks. Forty-two days later, the mice were sacrificed by CO2 administration, necropsies were performed. The tumors were weighted and cut into two parts. One half of the tumor was embedded in paraffin and subjected to TUNEL and immunohistochemical staining, the other half was frozen in liquid nitrogen for preparation. Tumor volume was calculated with using this eq. V (mm^3^) = *A* × *B*^2^/2, in which A is the largest diameter, and B is the vertical diameter.

### Flow cytometric detection of apoptosis

Apoptosis was detected using the annexin v-fluorescein isothiocyanate (FITC) apoptosis assay kit (oncogenic gene research product, Boston, MA) in the light of the manufacturer’s instructions. All samples were tested in three portions.

### Immunohistochemistry assay

Paraffin-embedded tumor tissues were used for PCNA immunostaining. After antigen retrieval, tissue sections were incubated with rabbit anti-human PCNA monoclonal antibody (Santa Cruz Biotechnology, CA, USA) for 30 min. After washing, second antibody was then added and incubated for 30 min (Dako cell death in Denmark). The negative control group was set with rabbit serum.

### TUNEL assay

Apoptosis of transplanted tumor tissues was detected by TUNEL kit (KeyGen, Nanjing, China) according to the manufacturer’s protocol.

### Luciferase activity

NF-κB-dependent luciferase reporter plasmid (2 × NF-κB-Luc) was constructed and maintained in our lab. After 30 h of transfection, the luciferase activity assay was performed using the Luciferase Activity Assay Kit (Promega, USA). After 48 h, the assay was operated again. The relative activity of luciferase is generally calculated by normalizing the ratio of firefly/renal luciferase to negative control transfected luciferase.

### Statistical analysis

Data were obtained from at least three independent experiments as noted. Student’s *t*-test (two-tailed) was used to compare the two groups. Statistics was performed in GraphPad Prism 6.0 software (San Diego, CA, USA). *p* < 0.05 indicated a statistically significant difference.

## Results

### Establishment and characterization of radioresistant HCC cell lines

**Fig. 1:**
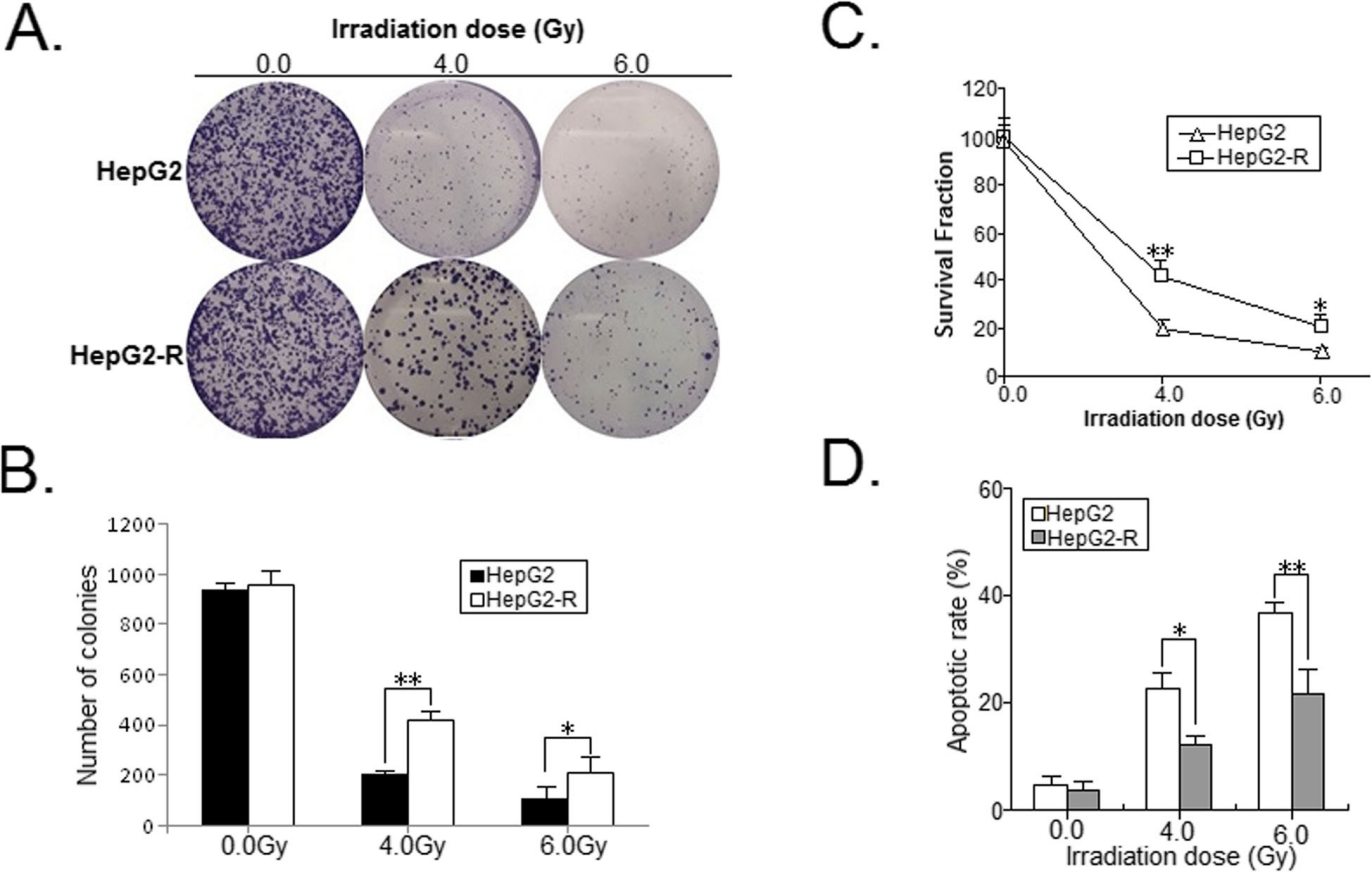
Establishment of radioresistant HCC cells. **a** A representative image of colony formation in parental and their radioresistant HCC cells treated with various dose of IR (0.0, 4.0 and 6.0 Gy) after 14 days. **b** The results of colony formation. **c** Survival fractions of parental and their radioresistant HCC cells were obtained from the results of the colony-forming assays. **d** Flow cytometric detection of apoptosis in parental and their radioresistant HCC cells treated with various dose of IR (0.0, 4.0 and 6.0 Gy). Data represent the mean ± S.E. of three individual experiments with triplicates. *p < 0.05 and**p < 0.01

In this study, we established a radioresistant HCC cell lines (HepG2-R) from parental HepG2 cell line. To establish a radioresistant HCC cell line, we exposed HepG2 cells to a range of radiation doses (0, 2.0, 4.0, 6.0, 8.0 and 10.0 Gy) over a period of 8 months. To further testify the radioresistant phenotype, we irradiated those parental and radioresistant HCC cells (0.0, 4.0 and 6.0 Gy) and examined them by colony formation assay. As shown in Fig. 1a-c, more foci formation and higher survival fractions in HepG2-R cells could be obtained when exposed to IR, compared with parental HepG2 cells. Also, flow cytometry was performed to detect the changes of apoptosis. As shown in Fig. 1d, HepG2-R cells showed more anti-apoptotic ability induced by IR, compared to parental HepG2 cells. These results indicated that HepG2-R cells had indeed acquired radioresistance.

### Aurora-a reduces the in vitro radiosensitivity of HCC cells by decreasing irradiation-induced apoptosis

**Fig. 2:**
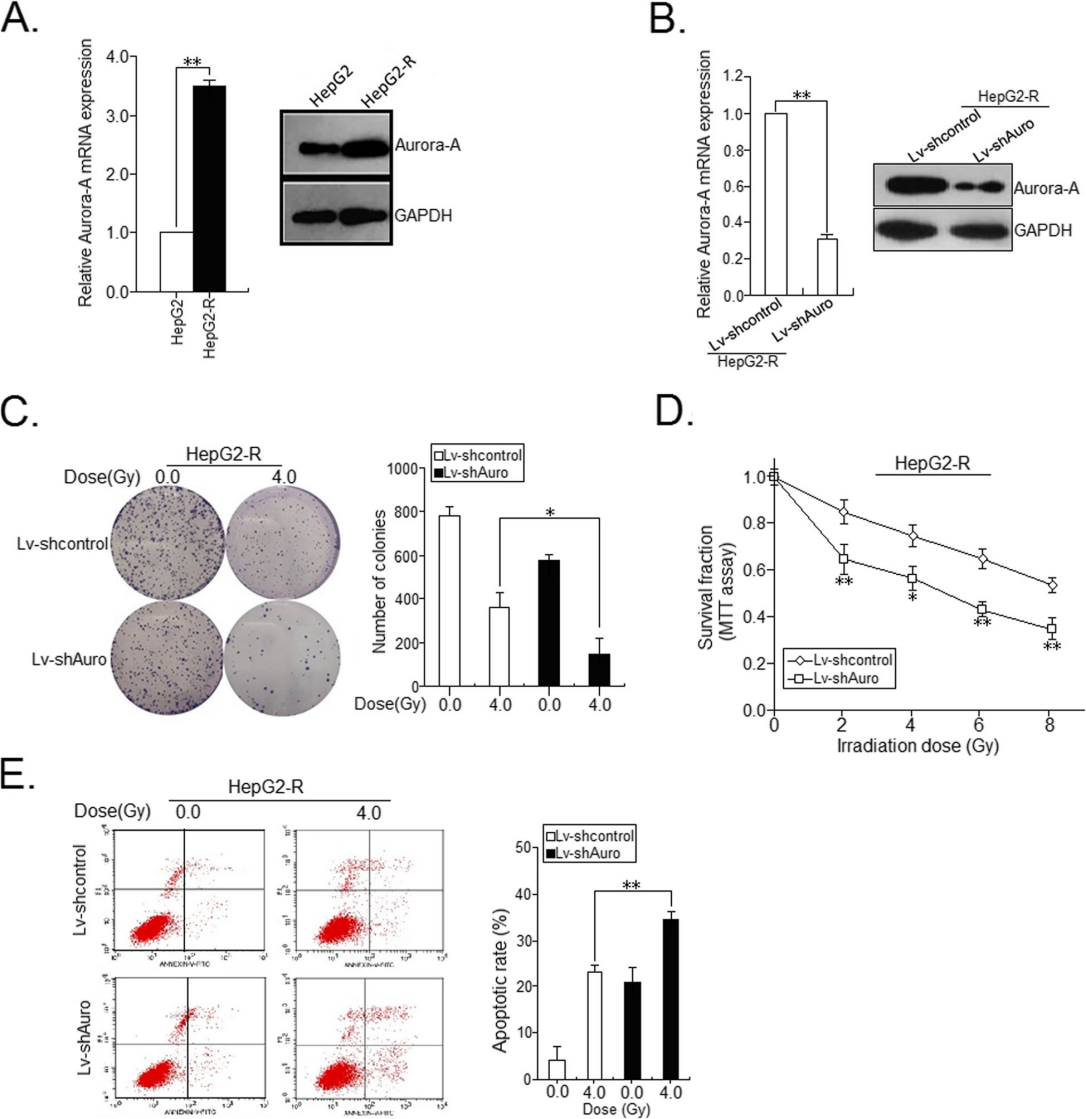
Effect of Aurora-A knockdown on in vitro radiosensitivity of radioresistant HCC cells. **a** RT-PCR and Western blot was used to detect the Aurora-A mRNA and protein expression in radioresistant and their parental HCC cells. The internal control was GAPDH. **b** RT-PCR and Western blot was used to detect the Aurora-A mRNA and protein expression in the stably transfected HCC cells (HepG2-R/shAuro or HepG2-R/shcontrol). The internal control was GAPDH. **c** A representative image of colony formation in the stably tansfected HCC cells treated with various dose of IR (0.0 and 4.0 Gy) after 14 days. **d** Survival fractions of the stably transfected HCC cells were obtained from the results of the MTT assays. **e** Flow cytometric detection of apoptosis in the stably transfected HCC cells treated with various dose of IR (0.0 and 4.0Gy). Data represent the mean ± S.E. of three individual experiments with triplicates. *p < 0.05 and**p < 0.01

**Fig. 3:**
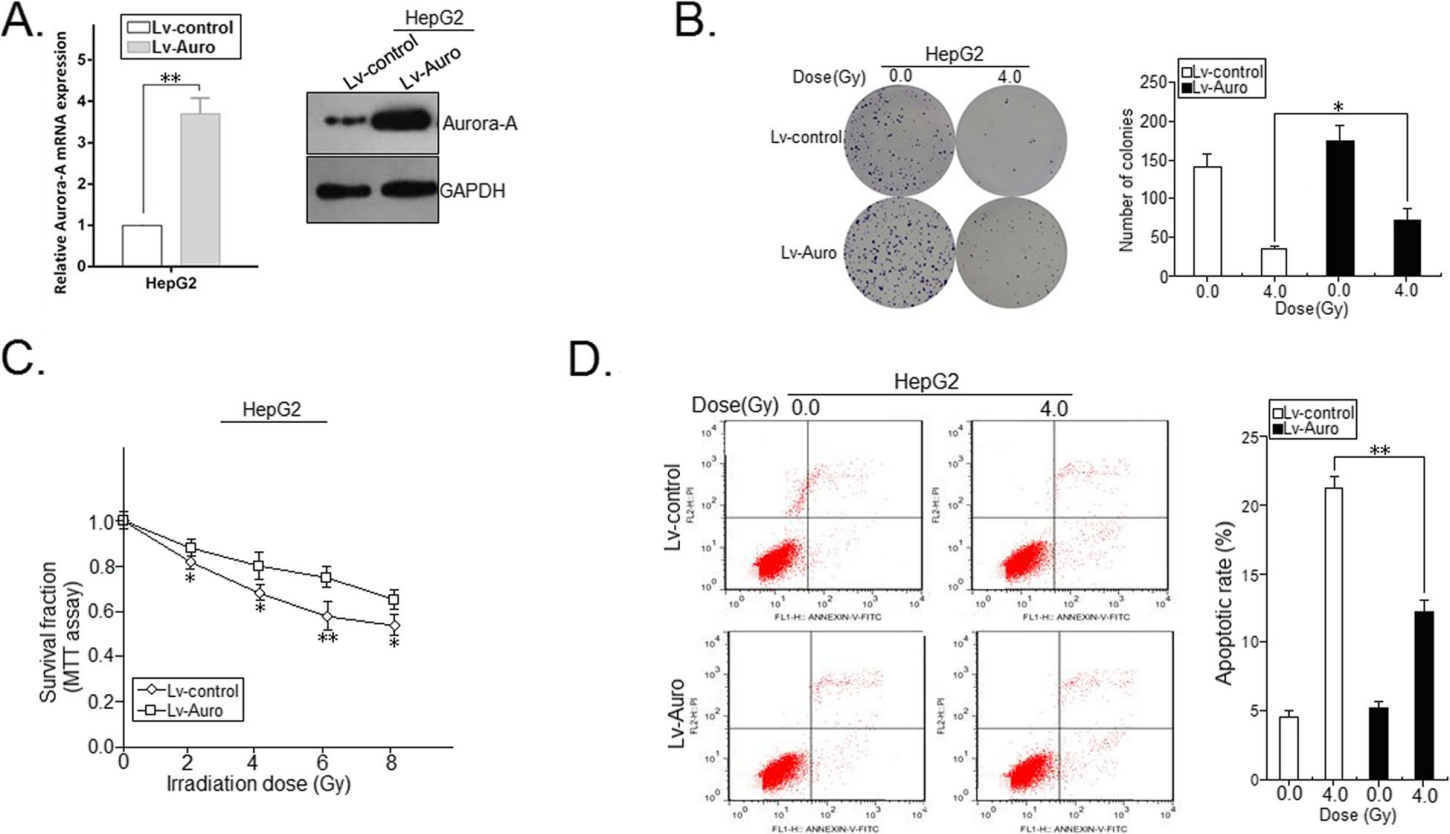
Effect of Aurora-A upregualtion on in vitro radiosensitivity of parental HCC cells. **a** RT-PCR and Western blot detection of Aurora-A mRNA and protein expression in the stably transfected HCC cells (HepG2/Auro or HepG2/control). The internal control was GAPDH. **b** A representative image of colony formation in the stably transfected HCC cells treated with various dose of IR (0.0 and 4.0 Gy) after 14 days. **c** Survival fractions of the stably transfected HCC cells were obtained from the results of the MTT assays. **d** Flow cytometric detection of apoptosis in the stably transfected HCC cells treated with various dose of IR (0.0 and 4.0Gy). Data represent the mean ± S.E. of three individual experiments with triplicates. *p < 0.05 and**p < 0.01

Previously, we have reported that overexpression of Aurora-A correlates with poor prognosis of HCC patients and its downregulation could induce growth inhibition and apoptosis enhancement in HCC cells [9, 10]. Also, we showed that Aurora-A promotes chemoresistance in HCC cells by targeting miR-21 [11]. However, whether Aurora-A plays important roles in HCC radioresistance is still unclear and remains to be further elucidated. qRT-PCR and Western blot assays were performed to detect the expression of Aurora-A mRNA and protein in radioresistant HCC cells and their parental HCC cells, and results showed that the expression levels of Aurora-A mRNA and protein in HepG2-R cells were significantly higher than those in HepG2 cells, respectively (Fig. 2a), suggesting that upregulation of Aurora-A plays a role in the development of HCC radioresistance. To further determine the roles of Aurora-A in HCC radioresistance, HepG2 (or HepG2-R) cells were stably transfected with lentiviral vector Lv-Auro (or Lv-control) or Lv-shAuro (or Lv-shcontrol). Then, qRT-PCR and Western blot assays confirmed the downregulation of Aurora-A in HepG2-R/shAuro cells and upregulation of Aurora-A in HepG2/Auro cells (Fig. 2b; Fig. 3a). The transfected HCC cells were treated with IR (4.0Gy), the effect of Aurora-A expression on colony formation of HCC cells was analyzed. Compared with that of HepG2-R/shcontrol cells combined with IR treatment (4.0Gy), the capacity of colony formation was significantly reduced in HepG2-R/shAuro cells combined with IR treatment (4.0Gy; Fig. 2c). Meanwhile, compared with that of HepG2/control cells, the capacity of colony formation was moderately increased in HepG2/Auro cells combined with IR treatment (4.0Gy; Fig. 3b). In addition, MTT assay was performed to measure the survival of those transfected cells with doses of IR (0.0, 2.0, 4.0, 6.0 and 8.0Gy). Knockdown of Aurora-A significantly increased the radiosensitivity of HepG2-R cells (Fig. 2d), while upregulation of Aurora-A reduced the radiosensitivity of HepG2 cells (Fig. 3c). Induced apoptosis is an important principle of radiotherapy. Thus, we examined the effect of Aurora-A expression on the IR-induced apoptosis of HCC cells. Flow cytometry analysis showed that silencing of Aurora-A increased irradiation-induced apoptosis of HepG2-R cells (4.0Gy; *p* < 0.01; Fig. 2e), whereas upregulation of Aurora-A reduced IR-induced apoptosis of parental HepG2 cells (4.0Gy; *p* < 0.01; Fig. 3d). The above data suggest that upregulation of Aurora-A reduces the radiosensitivity of HCC in vitro through decreasing IR-induced apoptosis.

### Aurora-a promotes the in vivo radioresistance of HCC cells

**Fig. 4:**
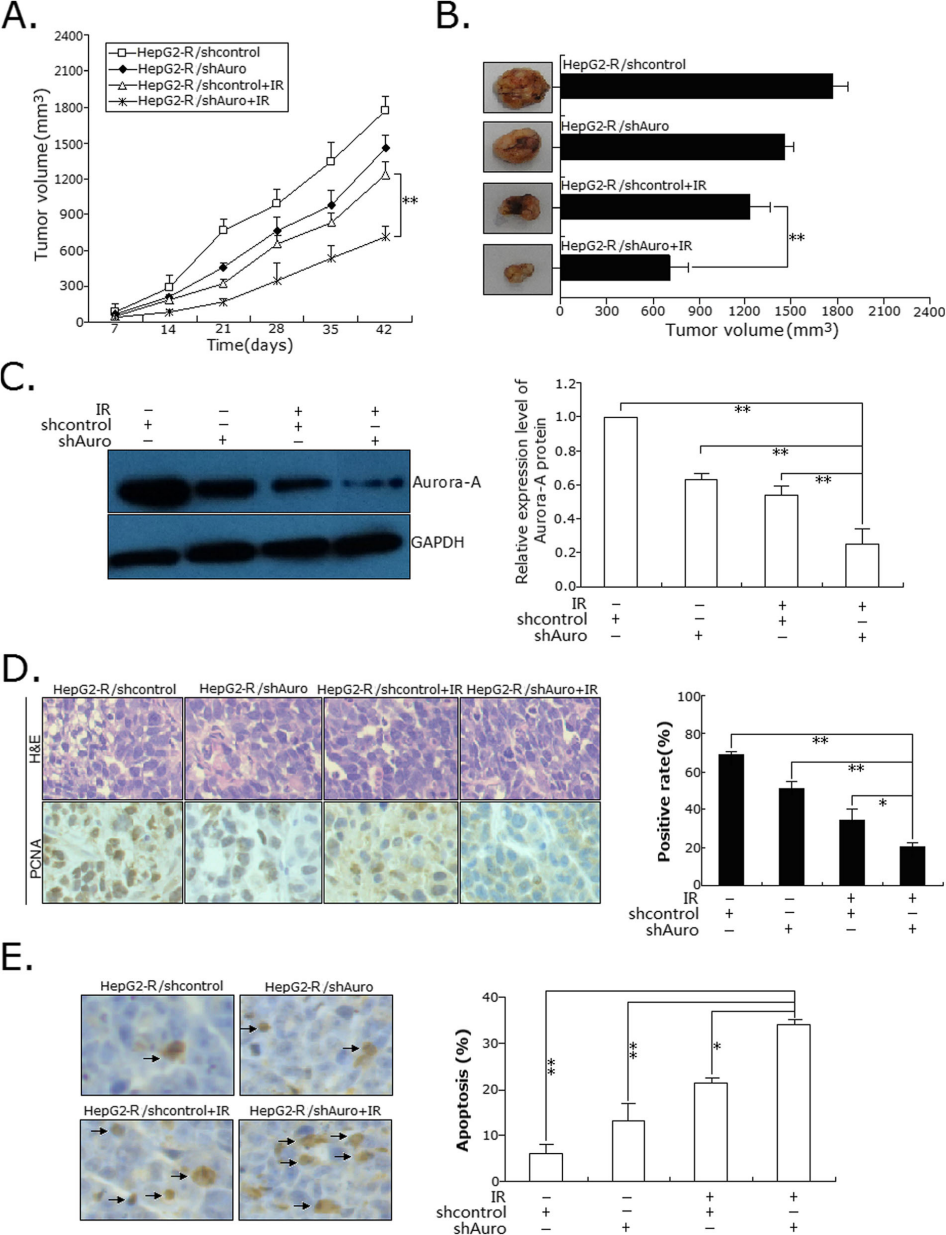
Effect of Aurora-A knockdown on in vivo radiosensitivity of radioresistant HCC cells. **a** The growth of subcutaneous tumor derived from HepG2-R/shAuro and HepG2-R/shcontrol cells in BALB/c athymic nude mice. Mice were treated with 8.0 Gy irradiation at seventh day post tumor cell injection. Five mice were inoculated. **b** Representative features of tumors 42d after inoculation using HepG2-R/shAuro and HepG2-R/shcontrol cells treated with IR. **c** Western blotting was used to detect the Aurora-A protein expression in tumors developed from HepG2-R/shAuro and HepG2-R/shcontrol cells treated with IR, respectively. The internal control was GAPDH. **d** Immunostaining of PCNA protein expression in tumors developed from HepG2-R/shAuro and HepG2-R/shcontrol cells treated with IR. Lower: immunostaining; Upper: H&E staining; Bars, 100 μm. **e** TUNEL assay was used to detect the apoptosis in tumors developed from HepG2-R/shAuro and HepG2-R/shcontrol cells treated with IR, respectively. Data represent the mean ± S.E. of three individual experiments with triplicates. *p < 0.05 and**p < 0.01

**Fig. 5:**
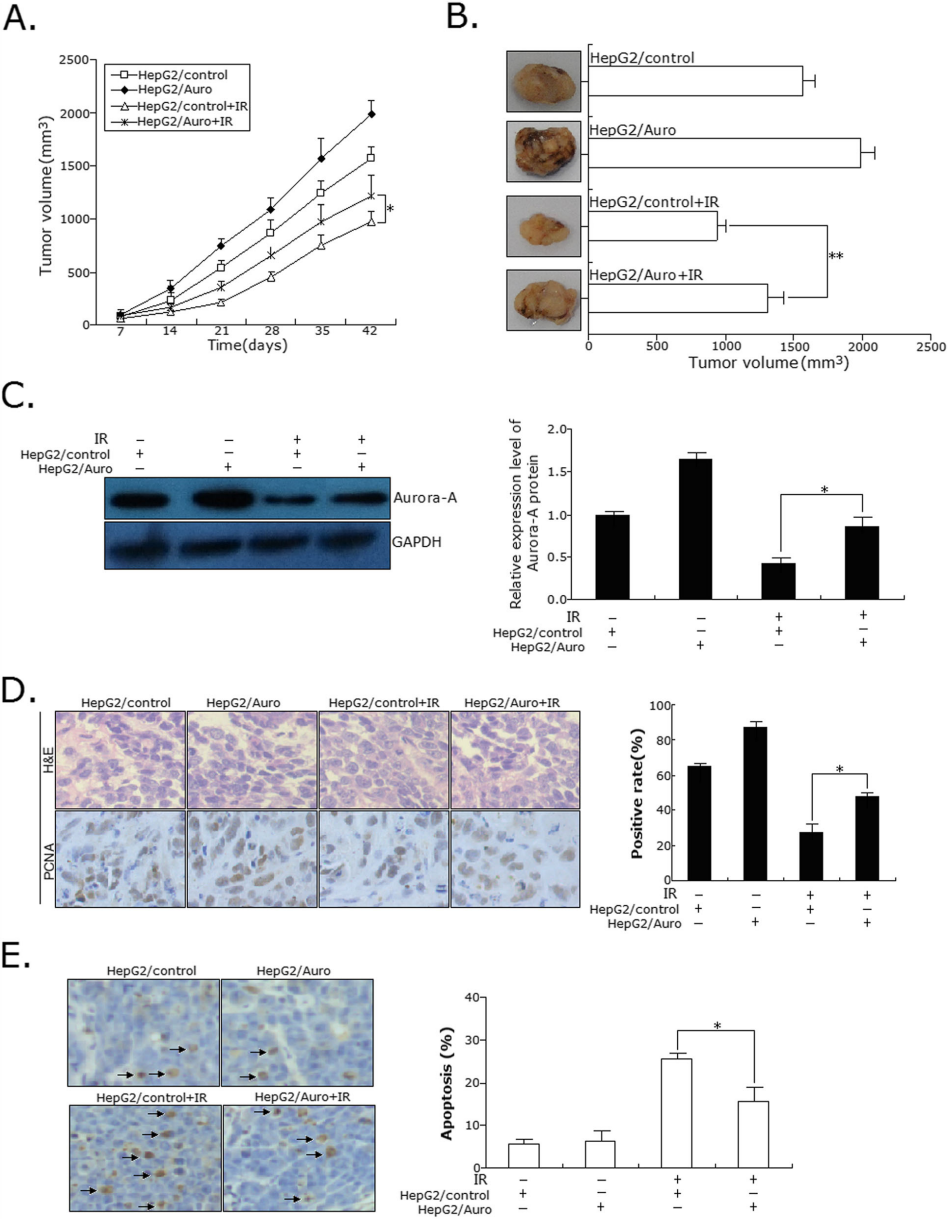
Effect of Aurora-A upregulation on in vivo radiosensitivity of parental HCC cells. **a** The growth of subcutaneous tumor derived from HepG2/Auro and HepG2/control cells in BALB/c athymic nude mice. Mice were treated with 8.0Gy irradiation at seventh day post tumor cell injection. Five mice were inoculated. **b** Representative features of tumors 42d after inoculation using HepG2/Auro and HepG2/control cells treated with IR. **c** Western blotting was used to detect the Aurora-A protein expression in tumors developed from HepG2/Auro and HepG2/control cells treated with IR, respectively. The internal control was GAPDH. **d** Immunostaining of PCNA protein expression in tumors developed from HepG2/Auro and HepG2/control cells treated with IR. Lower: immunostaining; Upper: H&E staining; Bars, 100 μm. **e** TUNEL assay was used to detect the apoptosis in tumors developed from HepG2/Auro and HepG2/control cells treated with IR, respectively. Data represent the mean ± S.E. of three individual experiments with triplicates. *p < 0.05 and**p < 0.01

To investigate the effect of Aurora-A expression on the vivo radiosensitivity of HCC cells, we generated subcutaneous tumors in nude mice using the stably transfected HepG2 cells [HepG2/control (or HepG2/Auro) or HepG2-R/shcontrol (or HepG2-R/shAuro)]. The xenografted mice were treated with IR at seventh day post tumor cell injection. After treated with IR, tumors developed more slowly in mice bearing the HepG2-R/shAuro xenograft than the control group (HepG2-R/shcontrol) (Fig. 4a), while tumors developed faster in mice bearing the HepG2/Auro xenograft than the control group (HepG2/control) (Fig. 5a). At 42 days after inoculation with or without IR treatment, the tumor volume was measured. The average volume of tumors formed from HepG2-R/shAuro cells was significantly lower than that formed from HepG2-R/shcontrol cells with IR treatment (*p* < 0.01; Fig. 4b), while the average volume of tumors formed from HepG2/Auro cells was significantly higher than that formed from HepG2/control (*p* < 0.01; Fig. 5b). Following IR treatment, tumor homogenates were subjected to Western blot detection of Aurora-A protein expression. Compared with xenografts formed from HepG2-R/shcontrol cells, the expression of Aurora-A protein was significantly downregulated in HepG2-R/shAuro cells. (Fig. 4c). Compared with xenografts formed from HepG2 /control cells, the expression of Aurora-A protein was significantly upregulated in HepG2/Auro cells. (Fig. 5c). Additionally, immunohistochemistry was performed to detect the expression of PCNA. The number of PCNA-positive cells in xenografts formed from HepG2-R/shAuro cells were higher than that in xenografts from HepG2-R/shcontrol cells (*p* < 0.05; Fig. 4d), while the number of PCNA-positive cells in xenografts formed from HepG2/Auro cells were lower than that in HepG2/control cells (*p* < 0.05; Fig. 5d). Furthermore, TUNEL assay was performed to detect the changes of apoptosis. The rate of apoptotic tumor cells in xenografts formed from HepG2-R/shAuro cells was higher than that in HepG2-R/shcontrol cells (*p* < 0.01; Fig. 4e), while the rate of apoptotic tumor cells in xenografts formed from HepG2/Auro cells were lower than that in HepG2/control cells (*p* < 0.01; Fig. 5e). These results suggest that Aurora-A promotes the in vivo radioresistance of HCC cells.

### Activation of NF-κB signaling is involved in Aurora-A-mediated HCC radioresistance

**Fig. 6:**
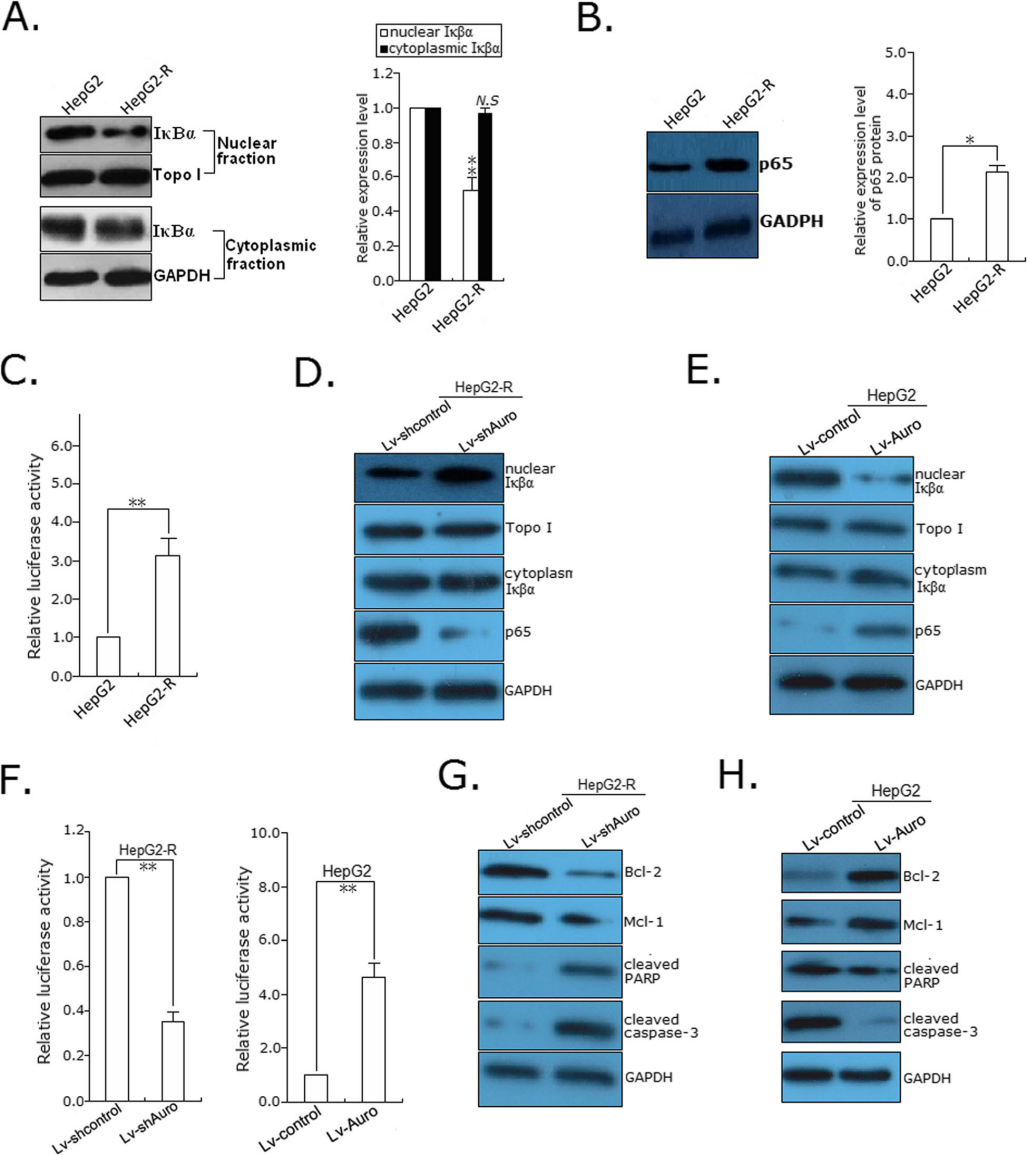
Aurora-a promotes the activation of NF-κB signaling in HCC cells. **a** Western blotting was used to detect the nuclear or cytoplasmic IκBα protein expression in parental and their radioresistant HCC cells. The internal control was GAPDH or Topo I, respectively. **b** Western blotting detection of p65 protein expression in parental and their radioresistant HCC cells. The internal control was GAPDH. **c** A luciferase reporter system was used to measure the activity of NF-κB in parental and their radioresistant HCC cells. **d** Western blotting was used to detect the nuclear or cytoplasmic IκBα protein and p65 expression in the Aurora-A-knockdown HCC cells. The internal control was GAPDH or Topo I, respectively. **e** Western blotting was used to detect the nuclear or cytoplasmic IκBα protein and p65 expression in the Aurora-A-overexpressing HCC cells. The internal control was GAPDH or Topo I, respectively. **f** A luciferase reporter system was used to measure the activity of NF-κB in the stably transfected HCC cells. **g** and **h** Western blotting was used to detect the apoptosis-related proteins (Bcl-2, Mcl-1, cleaved PARP and caspase-3) in the Aurora-A-knockdown and Aurora-A-overexpressing HCC cells. The internal control was GAPDH. Data represent the mean ± S.E. of three individual experiments with triplicates. *N.S, p* > 0.05, *p < 0.05 and**p < 0.01

Activation of NF-κB signaling has been reported to play a role in tumor radioresistance. Previous studies have shown that Aurora-A induces phosphorylation of IκBα, thereby mediating its degradation and loss of IκBα leads to activation of NF-κB target gene transcription [16]. Thus, we hypothesized that Aurora-A might promote HCC radioresistance by inducing NF-κB activation. NF-κB is present as a homodimer or a heterodimer with members of the Rel protein family. The distribution and function of p50/p65 are most extensive, but only the terminus of p65 contains a transactivation domain and activates gene transcription; thus, it is the major component of active forms of NF-κB. Therefore, p65 protein serves as the indicator of NF-κB. First, we detected the expression of IκBα and p65 proteins in the cytoplasmic and nuclear fractions of HCC cells by Western blot. The expression levels of nuclear IκBα protein were significantly decreased in HepG2-R cells in comparison with their parental cells (Fig. 6a), while the expression levels of nuclear p65 protein were significantly increased in those radioresistant HCC cells in comparison with their parental cells (Fig. 6d). Then, those parental and radioresistant HCC cells were transfected with a NF-κB-dependent luciferase reporter plasmid (2 × NF-κB-Luc), and assays of luciferase activity in lysates indicated that the NF-κB activity was significantly increased in HepG2-R cells in comparison with their parental cells (Fig. 6c). Next, we determined the effects of Aurora-A expression on the expression of p65 and IκBα in HCC cells, and demonstrated that knockdown of Aurora-A could result in the increased expression level of nuclear IκBα protein and the decreased expression of nuclear p65 protein in HepG2-R cells (Fig. 6d) and upregulation of Aurora-A could result in the decreased expression level of nuclear IκBα protein and the increased expression level of nuclear p65 protein in HepG2 cells (Fig. 6e). Also, those stably transfected HCC cells were transfected with a NF-κB-dependent luciferase reporter plasmid together, and assays of luciferase activity in lysates showed that knockdown of Aurora-A reduced NF-κB activity in radioresistant HCC cells and upregulation of Aurora-A increased the activity in their parental cells, while compared with control cells (Fig. 6f). Moreover, we assessed protein expressions of NF-κB downstream effectors containing Bcl-2, Mcl-1, cleaved PARP and cleaved caspase-3. Knockdown of Aurora-A in HepG2-R cells downregulated the protein levels of Bcl-2 and Mcl-1 and upregulated the protein levels of cleaved PARP and caspase-3 (Fig. 6g). Upregulation of Aurora-A in HepG2 cells contributed to the contrary results (Fig. 6h). It was strongly suggested that Aurora-A induced the activation of NF-κB signaling in HCC.

**Fig. 7:**
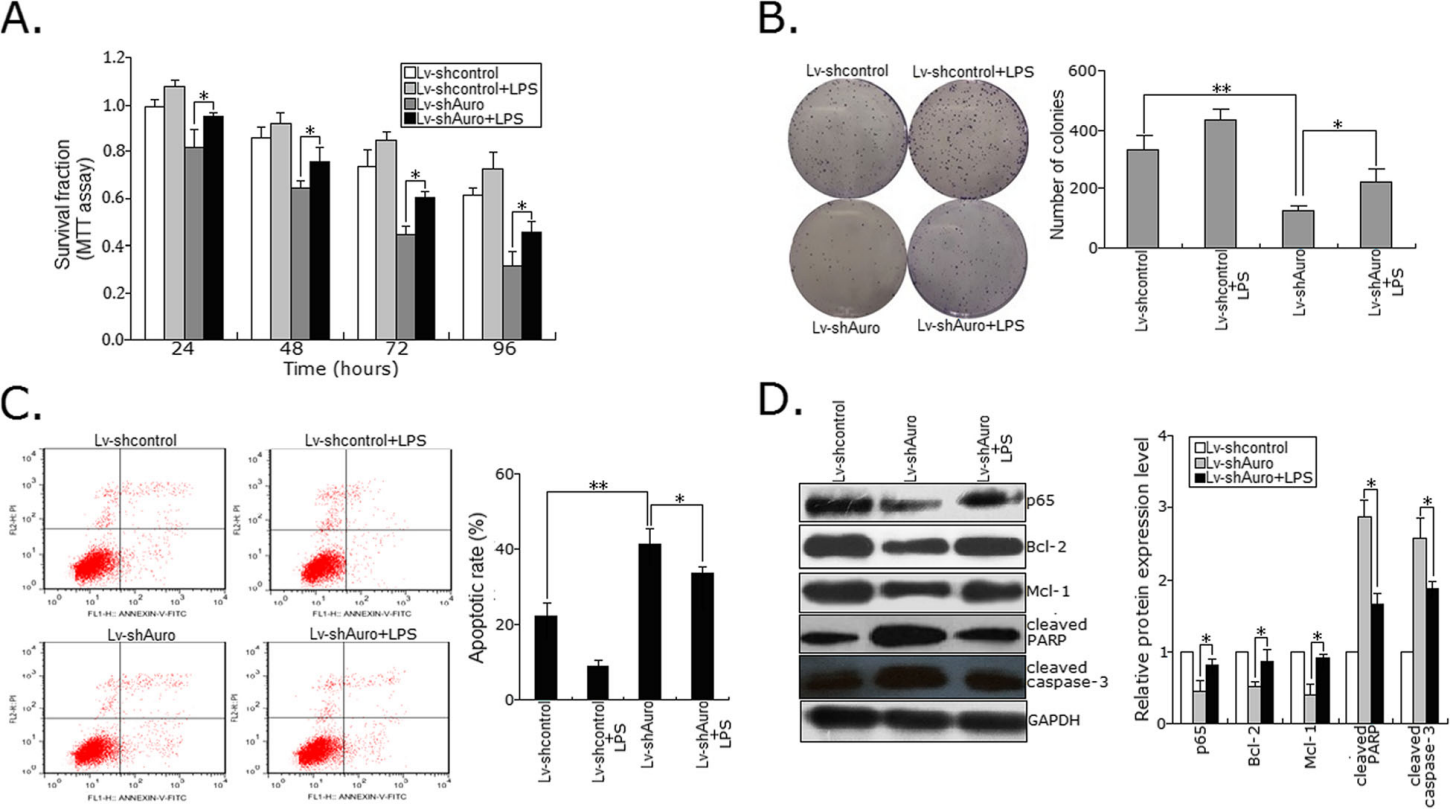
NF-κB signaling was involved in Aurora-A-promoting radioresistance in HCC cells. **a** MTT assays were employed to evaluate survival fractions of the stably transfected HCC cells plus LPS and IR (4.0Gy). **b** A representative image of colony formation in the stably transfected HCC cells plus LPS and IR (4.0Gy) after 14 days. **c** Flow cytometric detection of apoptosis in the stably transfected HCC cells plus LPS and IR (4.0Gy). **d** Western blot detection of the expression of those proteins (p65, Bcl-2, Mcl-1, cleaved PARP and caspase-3) in the stably transfected HCC cells plus LPS. GAPDH was used as an internal control. Data represent the mean ± S.E. of three individual experiments with triplicates. *p < 0.05 and**p < 0.01

To further detect the role of activation of NF-κB signaling in Aurora-A-mediated HCC radioresistance, LPS (an activator of NF-κB/p65) was co-incubated with HepG2-R cells infected with Lv-shAuro (or Lv-shcontrol). All those cells were treated with IR (4.0Gy), and then, the capacities of growth and colony formation and apoptosis were evaluated by MTT, colony formation and flow cytometry assays. Addition of LPS could partially reverse the decreased capacities of growth and colony formation and increased apoptosis in HepG2-R cells induced by Aurora-A knockdown (Fig. 7a-c). Also, we detected the expression of p65 protein and its downstream effectors. Addition of LPS could partially reverse the decreased expression of p65 protein and its downstream effectors (Bcl-2 and Mcl-1) and the increased expression of its downstream effectors (cleaved caspase-3 or PARP) (Fig. 7d). These data suggested that activation of NF-κB played a role in Aurora-A-promoted radioresistance in HCC cells.

## Discussion

As a major member of a new serine/threonine kinase family, Aurora-A has been reported to be involved in accurate bipolar spindle assembly, mitotic entry, separation of centriole pairs, and completion of cytokinesis and alignment of metaphase chromosomes [17]. Overexpression of Aurora-A could result in genetic instability, which could lead to malignant transformation of many tissues [18]. Aurora-A overexpression occurs in many human cancers, and this overexpression was associated with the patient’s prognosis. Landen’ et al. [19] indicated that Aurora-A was overexpressed by most of ovarian cancers and was related to centrosome amplification and poor survival. Tanaka et al. [20] showed that the malignant behavior of ESCC could be reflected by the up-regulated expression of Aurora-A, which could be used as a predictor in patients with ESCC. In addition, the relations between the overexpression of Aurora-A and the poor prognosis were also reported in other human malignancies, such as bladder cancer, breast cancer, and laryngeal cancer, etc. [21–23]. The relationship between overexpression of Aurora-A in HCC patients was first reported by Jeng and his colleagues. In their article, factors that affected the overexpression of Aurora-A were also mentioned, including high-grade and high-stage patients [8]. Our team’s former studies have demonstrated Aurora-A mRNA expression was highly relatived to advanced tumor stage and poorer prognosis of HCC patients [9]. In the latter study, we also analyzed the importance of the clinicopathology and prognostic of Aurora-A protein in HCC patients and indicated that the expression of Aurora-A was highly correlated with TNM staging and the lymph nodes metastasis. Survival analysis showed that HCC patients tended to have better overall survival with low Aurora-A protein expression in the contrast of patients with high Aurora-A protein expression. Multivariate Cox model analysis showed the increase of Aurora-A protein expressions were markers independent of regulatory factors in the overall survival rate of HCC patients, proposing Aurora-A might be a prognostic-marker for TNM staging of HCC. Therefore, the prognosis of HCC patients was negatively correlated with the overexpression of Aurora-A. In clinical practice, it could be selectively used to confirm the probability of HCC recurrence.

It has been proved by many studies that Aurora-A could regulate the malignant phenotype of a variety of tumor cells, such as growth, apoptosis, invasion and metastasis, chemoresistance, etc. Wang’ et al. [24] showed that Aurora-A could promote ESCC cell proliferation and prolong apoptosis. The same group indicated that RNA interfered with human ESCC cell line, stably down-regulated Aurora-A, inhibited tumor cell proliferation, shortened apoptosis time, and provided a promising therapeutic strategy to treat ESCC [25]. In addition, Tanaka’ et al. [26] indicated that targeting Aurora-A inhibited the growth of human OSCC cells in vitro and in vivo. Also, it was reported that Aurora-A promoted invasion and metastasis in a variety of human cancers. For example, Maimaiti’ et al. [27] found that Aurora-A induced lymph node metastasis of papillary thyroid carcinoma by promoting cofilin-1 activity. Wang’ et al. [28] showed that Aurora-A activated the Cofilin-F-actin pathway inducing mammary cell migration and breast cancer metastasis. Importantly, Aurora-A was reported to regulate tumor chemoresistance. Sun’ et al. [29] showed that inhibition of Aurora-A promoted chemosensitivity via inducing cell cycle arrest and apoptosis in cervical cancer cells. Also, it was reported that Aurora-A could induce cell survival and chemoresistance by activation of Akt through a p53-dependent manner in ovarian cancer cells [30]. Meanwhile, the correlation of tumor radioresistance with Aurora-A expression was also studied. Ma’ et al. showed that Aurora-A affected radiosensitivity in cervical squamous cell carcinoma [31]. Venkataraman’ et al. [32] showed that targeting Aurora Kinase A enhanced radiation sensitivity of atypical teratoid rhabdoid tumor cells. The above data indicated that Aurora-A might be a molecular target for chemosensitizing or radiosensitizing human tumors. In the former study, we have concluded that RNA interference suppressed proliferation and induced apoptosis in human HCC cells through targeting Aurora-A. Our further research showed that Aurora-A activating Akt and p38-MAPK signaling pathways might be a key regulator of HIF-1α-promoting malignant phenotypes of HCC [33]. Meanwhile, we showed that methylation-associated silencing of microRNA-129-3p might target Aurora-A to regulate epithelial-mesenchymal transition, invasion, and metastasis of HCC. Furthermore, we testified that Aurora-A could promote HCC chemoresistance by targeting NF-kappaB/microRNA-21/PTEN signaling pathway [11]. In another report, Benten’ et al. [34] indicated that the growth of HCC was suppressed by Aurora kinase inhibitor PHA-739358 in vitro and in vivo. Although HCC progression is significantly related to Aurora-A overexpression, the relationship between the expression of Aurora-A and the HCC radioresistance remains unclear. To prove it, we successfully established one radioresistant HCC cell line from its parental cell line and showed that Aurora-A was significantly upregulated in radioresistance HCC cells in comparison with their parental cells. Then, we performed functional assays. Knockdown of Aurora-A could reverse radioresistance of radioresistant HCC by increasing radiotherapy-induced apoptosis, while upregulation of Aurora-A reduced radiosensitivity of HCC cells by decreasing radiotherapy-induced apoptosis. Consequently, up-regulation of Aurora-A promoted the formation of radioresistance in HCC.

Furthermore, we explored the molecular mechanisms of Aurora-A promoting radioresistance in HCC cells. Previous studies have shown overexpression of Aurora-A might enhance the activity of NF-κB. Chefetz’ et al. [35] showed that inhibition of Aurora-A kinase induced cell cycle arrest by affecting NF-κB pathway in epithelial ovarian cancer stem cells. In lung cancer cells with p53 gene silencing, the study found that Aurora-A could promote the resistance of gefitinib with NF-κB signaling pathway [36]. Also, Linardopoulos and his colleagues indicated that Aurora-A-inhibition enhanced the efficacy of chemotherapy drugs and acquires resistance from activated NF-κB [37]. NF-κB plays an important role in carcinogenesis as well as in the regulation of inflammatory and immune responses. NF-κB induces the expression of varying target genes, which has been related to various cellular processes in cancer, including inflammation, proliferation, angiogenesis, transformation, invasion, and metastasis [38]. Meanwhile, activation of NF-κB contributes to resistance to chemotherapy and ionizing radiation during cancer treatment. It has been reported that irradiation activated complex anti-apoptotic and other transcription factors so that cancer cells could not repair DNA damage and obtained anti-apoptotic radiation therapy. Therefore, in most cell types, NF-κB is recognized as a type of key that protects cells from apoptosis [39]. Our and other studies have shown that Aurora-A activated nuclear factor-kappaB signaling by phosphorylation of IκBα. Although we previously reported that Aurora-A could promote HCC chemoresistance by targeting NF-κB signaling, whether Aurora-A promotes HCC radioresistance by activation this signaling is unclear. First, the expression levels of nuclear IκBα protein in radioresistant HCC cells were found to be lower than that in parental HCC cells, while the expression levels of p65 protein in radioresistant HCC cells were higher than that in parental cells. Meanwhile, the activity of NF-κB in radioresistant HCC cells was stronger than that in parental cells. Importantly, knockdown of Aurora-A might lead to an increase in the expression of nuclear IκBα protein and the decreased expression of p65 protein in radioresistant HCC cells, while upregulation of Aurora-A could cause the contrary results in parental HCC cells. Moreover, we detected the expression of NF-κB pathway downstream effectors of Bcl-2, Mcl-2, cleaved PARP, and caspase-3. Knockdown of Aurora-A downregulated Bcl-2 and Mcl-1 protein expressions and upregulated cleaved PARP and caspase-3 expression in radioresistance. Upregulation of Aurora-A obtained the reverse results in parental HCC cells. Bcl-2, Mcl-2, PARP, and caspase-3 levels were closely linked to regulation in cell apoptosis, so the changes of downstream effectors expressions supported that NF-κB pathway contributed to Aurora-A-induced regulation of apoptosis in the formation of HCC radioresistance. In another report, Aurora-A was found to contribute to radioresistance by increasing NF-κB DNA binding activity. The system regulation of NF-κB pathway mediated by Aurora-A is not fully understood. Also, whether Aurora-A participates in HCC radioresistance by regulating other signaling pathways is still unclear. Therefore, further understanding of Aurora-A protein role in HCC radioresistance and regulation of molecular signaling pathways might provide new insights into the effective treatments in HCC.

## Conclusions

In summary, this study confirmed that Aurora-A contributes to HCC radioresistance through reducing radiotherapy-induced apoptosis by activating NF-κB signaling. This signaling pathway provides new targets for radiosensitization of HCC.

## Data Availability

All data generated or analysed during this study are included in this published article.

## Abbreviations

ESCC: Esophageal squamous cell carcinoma
FITC: Fluorescein isothiocyanate
GAPDH: Glyceraldehyde- 3-phosphate dehydrogenase
HCC: Hepatocellular carcinoma
MTT: 3-(4,5-Dimethyl-2-thiazolyl)-2, 5-diphenyl-2H-tetrazolium bromide
PBS: Phosphate buffer solution
PCNA: Proliferating Cell Nuclear Antigen
qRT-PCR: Quantitative real-time polymerase chain reaction
TUNEL: Dterminal dexynucleotidyl transferase (TdT)-mediated dUTP nick end labeling

## References

[1] Siegel RL, Miller KD, Jemal A (2018). Cancer statistics, 2018. CA Cancer J Clin.

[2] Pascual S, Herrera I, Irurzun J (2016). New advances in hepatocellular carcinoma. World J Hepatol.

[3] Kulik LM, Carr BI, Mulcahy MF, Lewandowski RJ, Atassi B, Ryu RK, Sato KT, Benson A, Nemcek AA, Gates VL (2008). Safety and efficacy of 90Y radiotherapy for hepatocellular carcinoma with and without portal vein thrombosis. Hepatology..

[4] Mornex F, Girard N, Beziat C, Kubas A, Khodri M, Trepo C, Merle P (2006). Feasibility and efficacy of high-dose three-dimensional-conformal radiotherapy in cirrhotic patients with small-size hepatocellular carcinoma non-eligible for curative therapies--mature results of the French phase II RTF-1 trial. Int J Radiat Oncol Biol Phys.

[5] Ducat D, Zheng Y (2004). Aurora kinases in spindle assembly and chromosome segregation. Exp Cell Res.

[6] Yan M, Wang C, He B, Yang M, Tong M, Long Z, Liu B, Peng F, Xu L, Zhang Y (2016). Aurora-A Kinase: a potent oncogene and target for cancer therapy. Med Res Rev.

[7] D’Assoro AB, Haddad T, Galanis E (2016). Aurora-A Kinase as a promising therapeutic target in cancer. Front Oncol.

[8] Jeng YM, Peng SY, Lin CY, Hsu HC (2004). Overexpression and amplification of Aurora-A in hepatocellular carcinoma. Clin Cancer Res.

[9] Wang R, Wang JH, Chu XY, Geng HC, Chen LB (2009). Expression of STK15 mRNA in hepatocellular carcinoma and its prognostic significance. Clin Biochem.

[10] Gao P, Wang R, Shen JJ, Lin F, Wang X, Dong K, Zhang HZ (2008). Hypoxia-inducible enhancer/alpha-fetoprotein promoter-driven RNA interference targeting STK15 suppresses proliferation and induces apoptosis in human hepatocellular carcinoma cells. Cancer Sci.

[11] Zhang K, Chen J, Chen D, Huang J, Feng B, Han S, Chen Y, Song H, De W, Zhu Z (2014). Aurora-A promotes chemoresistance in hepatocelluar carcinoma by targeting NF-kappaB/microRNA-21/PTEN signaling pathway. Oncotarget..

[12] McBride WH, Pajonk F, Chiang CS, Sun JR (2002). NF-kappa B, cytokines, proteasomes, and low-dose radiation exposure. Mil Med.

[13] Guttridge DC, Albanese C, Reuther JY, Pestell RG, Baldwin AS (1999). NF-kappaB controls cell growth and differentiation through transcriptional regulation of cyclin D1. Mol Cell Biol.

[14] Sonenshein GE (1997). Rel/NF-kappa B transcription factors and the control of apoptosis. Semin Cancer Biol.

[15] Chen C, Edelstein LC, Gélinas C (2000). The Rel/NF-kappaB family directly activates expression of the apoptosis inhibitor Bcl-x(L). Mol Cell Biol.

[16] Briassouli P, Chan F, Savage K, Reis-Filho JS, Linardopoulos S (2007). Aurora-A regulation of nuclear factor-kappaB signaling by phosphorylation of IkappaBalpha. Cancer Res.

[17] Kufer TA, Nigg EA, Silljé HH (2003). Regulation of Aurora-A kinase on the mitotic spindle. Chromosoma..

[18] Katayama H, Brinkley WR, Sen S (2003). The Aurora kinases: role in cell transformation and tumorigenesis. Cancer Metastasis Rev.

[19] Landen CN, Lin YG, Immaneni A, Deavers MT, Merritt WM, Spannuth WA, Bodurka DC, Gershenson DM, Brinkley WR, Sood AK (2007). Overexpression of the centrosomal protein Aurora-a kinase is associated with poor prognosis in epithelial ovarian cancer patients. Clin Cancer Res.

[20] Tanaka E, Hashimoto Y, Ito T, Okumura T, Kan T, Watanabe G, Imamura M, Inazawa J, Shimada Y (2005). The clinical significance of Aurora-a/STK15/BTAK expression in human esophageal squamous cell carcinoma. Clin Cancer Res.

[21] Mobley A, Zhang S, Bondaruk J, Wang Y, Majewski T, Caraway NP, Huang L, Shoshan E, Velazquez-Torres G, Nitti G (2017). Aurora Kinase A is a biomarker for bladder cancer detection and contributes to its aggressive behavior. Sci Rep.

[22] Zhang H, Chen X, Jin Y, Liu B, Zhou L (2012). Overexpression of Aurora-A promotes laryngeal cancer progression by enhancing invasive ability and chromosomal instability. Eur Arch Otorhinolaryngol.

[23] Nadler Y, Camp RL, Schwartz C, Rimm DL, Kluger HM, Kluger Y (2008). Expression of Aurora A (but not Aurora B) is predictive of survival in breast cancer. Clin Cancer Res.

[24] Wang XX, Liu R, Jin SQ, Fan FY, Zhan QM (2006). Overexpression of Aurora-a kinase promotes tumor cell proliferation and inhibits apoptosis in esophageal squamous cell carcinoma cell line. Cell Res.

[25] Wang X, Dong L, Xie J, Tong T, Zhan Q (2009). Stable knockdown of Aurora-a by vector-based RNA interference in human esophageal squamous cell carcinoma cell line inhibits tumor cell proliferation, invasion and enhances apoptosis. Cancer Biol Ther.

[26] Tanaka H, Nakashiro K, Iwamoto K, Tokuzen N, Fujita Y, Shirakawa R, Oka R, Goda H, Hamakawa H (2013). Targeting Aurora kinase a suppresses the growth of human oral squamous cell carcinoma cells in vitro and in vivo. Oral Oncol.

[27] Maimaiti Y, Jie T, Jing Z, Changwen W, Pan Y, Chen C, Tao H (2016). Aurora kinase A induces papillary thyroid cancer lymph node metastasis by promoting cofilin-1 activity. Biochem Biophys Res Commun.

[28] Wang LH, Xiang J, Yan M, Zhang Y, Zhao Y, Yue CF, Xu J, Zheng FM, Chen JN, Kang Z (2010). The mitotic kinase Aurora-a induces mammary cell migration and breast cancer metastasis by activating the Cofilin-F-actin pathway. Cancer Res.

[29] Sun JM, Yang LN, Xu H, Chang B, Wang HY, Yang G (2015). Inhibition of Aurora a promotes chemosensitivity via inducing cell cycle arrest and apoptosis in cervical cancer cells. Am J Cancer Res.

[30] Yang H, He L, Kruk P, Nicosia SV, Cheng JQ (2006). Aurora-a induces cell survival and chemoresistance by activation of Akt through a p53-dependent manner in ovarian cancer cells. Int J Cancer.

[31] Ma Y, Yang J, Wang R, Zhang Z, Qi X, Liu C, Ma M (2017). Aurora-a affects radiosenstivity in cervical squamous cell carcinoma and predicts poor prognosis. Oncotarget..

[32] Venkataraman S, Alimova I, Tello T, Harris PS, Knipstein JA, Donson AM, Foreman NK, Liu AK, Vibhakar R (2012). Targeting Aurora kinase a enhances radiation sensitivity of atypical teratoid rhabdoid tumor cells. J Neuro-Oncol.

[33] Cui SY, Huang JY, Chen YT, Song HZ, Huang GC, De W, Wang R, Chen LB (2013). The role of Aurora a in hypoxia-inducible factor 1α-promoting malignant phenotypes of hepatocelluar carcinoma. Cell Cycle.

[34] Benten D, Keller G, Quaas A, Schrader J, Gontarewicz A, Balabanov S, Braig M, Wege H, Moll J, Lohse AW (2009). Aurora kinase inhibitor PHA-739358 suppresses growth of hepatocellular carcinoma in vitro and in a xenograft mouse model. Neoplasia.

[35] Chefetz I, Holmberg JC, Alvero AB, Visintin I, Mor G (2011). Inhibition of Aurora-a kinase induces cell cycle arrest in epithelial ovarian cancer stem cells by affecting NFĸB pathway. Cell Cycle.

[36] Wu CC, Yu CT, Chang GC, Lai JM, Hsu SL (2011). Aurora-a promotes gefitinib resistance via a NF-κB signaling pathway in p53 knockdown lung cancer cells. Biochem Biophys Res Commun.

[37] Linardopoulos S (2007). Aurora-a kinase regulates NF-kappaB activity: lessons from combination studies. J Buon.

[38] Hoesel B, Schmid JA (2013). The complexity of NF-κB signaling in inflammation and cancer. Mol Cancer.

[39] Bai M, Ma X, Li X, Wang X, Mei Q, Li X, Wu Z, Han W (2015). The accomplices of NF-κB Lead to Radioresistance. Curr Protein Pept Sci.

